# Differential Gene Expression for *Curvularia eragrostidis* Pathogenic Incidence in Crabgrass (*Digitaria sanguinalis*) Revealed by cDNA-AFLP Analysis

**DOI:** 10.1371/journal.pone.0075430

**Published:** 2013-10-08

**Authors:** Jianshu Wang, Xuemin Wang, Bohua Yuan, Sheng Qiang

**Affiliations:** 1 Weed Research Laboratory, Nanjing Agricultural University, Nanjing, People’s Republic of China; 2 Agricultural College, Hebei University of Engineering, Handan, People’s Republic of China; Wuhan Bioengineering Institute, China

## Abstract

Gene expression profiles of *Digitaria sanguinalis* infected by *Curvularia eragrostidis* strain QZ-2000 at two concentrations of conidia and two dew durations were analyzed by cDNA amplified fragment length polymorphisms (cDNA-AFLP). Inoculum strength was more determinant of gene expression than dew duration. A total of 256 primer combinations were used for selective amplification and 1214 transcript-derived fragments (TDFs) were selected for their differential expression. Of these, 518 up-regulated differentially expressed TDFs were identified. Forty-six differential cDNA fragments were chosen to be cloned and 35 of them were successfully cloned and sequenced, of which 25 were homologous to genes of known function according to the GenBank database. Only 6 genes were up-regulated in *Curvularia eragrostidis*-inoculated *D. sanguinalis*, with functions involved in signal transduction, energy metabolism, cell growth and development, stress responses, abscisic acid biosynthesis and response. It appears that a few pathways may be important parts of the pathogenic strategy of *C.* *eragrostidis* strain QZ-2000 on *D. sanguinalis*. Our study provides the fundamentals to further study the pathogenic mechanism, screen for optimal *C. eragrostidis* strains as potential mycoherbicide and apply this product to control *D.* *sanguinalis*.

## Introduction

Large crabgrass (*Digitaria sanguinalis*), is an annual plant with a great reproductive capacity that almost all cutting nodes with fibrous roots and buds can develop into new plants [1]. Large crabgrass, one of the worst weeds in arid agricultural areas, has been reported as a weed infecting 33 crops and turfgrass in fifty-six countries [2], [3], throughout tropical and temperate regions of the world [4], [5]. Use of chemical herbicide is the major strategy for crabgrass control in most crops at present, however, intensive and repeated use of chemicals over several decades has led to the evolution of herbicide-resistant crabgrass [6], [7] and environmental pollution, therefore, biological control, if available, would be a preferable alternative for its control. Biological control agents, especially plant pathogenic microbes, have been shown effective in controlling some specific troublesome weeds. For example, *Ustilago syntherismae* and *Curvularia intermedia* have been reported as potential biological control agents of crabgrass [8], [9]. Mixture of three fungal pathogens *Drechslera gigantean*, *Exserohilum longirostratum*, and *Exserohilum rostratum* were evaluated as a potential biological control agent against large crabgrass and other weedy grasses [10]. Zhu and Qiang (2003) employed a strain of *Curvularia eragrostidis*, designated as QZ-2000, isolated from a naturally-occurring diseased plant of large crabgrass to biocontrol this weed [11]. Systematic evaluation of the pathogenicity, host specificity, and culture condition determined a good efficacy of *C. eragrostidis* when incubated at or above 2×10^5^ spore or more dosage and with dew for more than 24 hrs. This strain is safe to most crops and economic plants, so it has the potential to be developed into a mycoherbicide against large crabgrass [12]. But the pathogenic effects and molecular mechanisms remain unknown. In this study, effects of two spore concentrations and two dew durations on pathogenicity of QZ-2000 to *D. sanguinalis* were evaluated, and the differentially expressed genes of the infected plants were simultaneously detected by cDNA-AFLP. Forty-six differential cDNA fragments were chosen to cloned and 35 of them were successfully cloned and sequenced, of which 25 were homologous to genes of known function according to the GenBank database. Results obtained demonstrated that *C. eragrostidis* can infect, distort gene expression and ultimately damage *D. sanguinalis*. The main objectives are to build gene expression profiles during *C. eragrostidis* infection of *D. sanguinalis* and elucidate the mechanism in this fungus infection and killing of this weed.

## Materials and Methods

### Plant and Fungal Materials

Large crabgrass seeds were germinated on moistened filter paper in Petri dishes at 25°C for 3 days, twenty seedlings were planted into each plastic pot containing commercial potting medium. Plants were grown in the greenhouse, 30/25±5°C day/night temperature with of 12 h photoperiod. *C. eragrostidis* was cultured on potato dextrose agar (PDA) in 9-cm Petri dishes, and maintained at 25°C in the dark. Once vegetative hyphae fully covered the medium, the mycelia were exposed to black light (20 W, wavelength 300–380 nm, 100% humidity) for 48 h to induce sporulation [12]. To harvest conidia, autoclaved water was added to the cultures, which were gently shaken for 10 min. The conidia were collected by centrifugation at 1000 rpm (178×g) for 10 min, washed twice and diluted with distilled water to concentrations of 5×10^5^ and 1×10^6^ conidia/mL. Each conidia suspension was sprayed to four-leaf seedling of *D. sanguinalis* at 70 mL/m^2^. Inoculated and control seedlings were placed in a mist chamber for 12 and 24 h at 25°C. Non inoculated control plants received the same mist chamber treatment. The experiments were repeated three times with three pots per group.

### Assessment of Disease Incidence

Disease severity and differences among treatments were assessed by determining mortality and above-ground dry biomass per pot 10 days after inoculation. Dry weight (DW) was obtained by cutting the aerial parts at soil level, drying in the paper bags in an electric oven at 80°C for 40 h, and then weighing. Dry weight data were expressed as percent reduction in biomass compared with biomass of non inoculated controls.

### RNA Extraction and cDNA-AFLP Analysis

The aerial parts of the seedlings were collected for RNA extraction after 4 d inoculation. Total RNA was isolated from the frozen tissues according to a modified CTAB method described in Li et al. (2011) [13]. cDNA-AFLP analysis was carried out as described by Bachem [14]. Briefly, 5 µg of total RNA was converted to cDNA using oligo-dT primer and MMLV reverse transcriptase (Takara, Dalian). About 100 ng of synthesized double-strand cDNA was digested with EcoRI and MseI restriction enzyme (New England Biolabs). After digestion, the restriction fragments were ligated with the following adaptors: EcoRI-F, 5′-CTCGTAGACTGCGTACC-3′; EcoRI-R, 5′-AATTGGTACGCAGTC-3′; MseI-F, 5′-GACGATGAGTCCTGAG-3′; and MseI-R, 5′-TACTCAGGACTCAT-3′. Ligated cDNA fragments were subsequently amplified by PCR using nonselective EcoRI and MseI primers (EcoRI-F, 5′-GACTGCGTACCAATTC-3′; MseI-R, 5′-GATGAGTCCTGAGTAA-3′). This PCR reaction was carried out using 1 cycle of 95°C for 2 min followed 20 cycles of 95°C for 30 s, 50°C for 30 s, and 72°C for 1 min. The amplified cDNA was diluted 100-fold with distilled water and then was used for selective PCR amplification using selective EcoRI and MseI primers (EcoRI: 5′-GACTGCGTACCAATTCNN-3′ and MseI: 5′-GATGAGTCCTGAGTAANN-3′). The selective PCR cycles were performed by touchdown PCR methods as described by Vos *et al.* [15]. In total, 256 primer combinations were conducted for selective PCR amplifications. The PCR products were separated on a 4% denaturing polyacrylamide gel running with 80 W for 2.5 h. cDNA fragments were visualized by silver staining according to the protocol described by Bassam *et al.* [16].

### Data Scoring and Cluster Analysis

Data scoring and genetic similarity coefficient analyses were performed as described by Guo *et al*. [17]. The similarity coefficient matrix was subjected to cluster analysis by unweighted pair group method of arithmetic averages (UPGMA) and a dendrogram was generated using NTSYSpc software Version 2.0 (Exeter Software, New York, USA).

### Transcript-derived Fragment (TDF) Isolation

The bands of interest were selected, removed from the gel and soaked in 10 µL water, DNA was purified by precipitation and then reamplified using the same primers as for the selective amplification totaling 36 cycles, but touchdown conditions were carried out as follows: reduction of the annealing temperature from 65°C to 59°C, in 1.0°C steps, which was then maintained for 30 cycles. The PCR products were further separated by 5% nature polyacrylamide gel electrophoresis, the target bands were cut, purified, and reamplified again as described as above. The reamplified TDFs were cloned into plasmid pMD18-T through TA Cloning strategy (Takara) and sequenced. Database searches were performed using the BLAST Network Service (NCBI, National Center for Biotechnology Service). Each TDF sequence was compared against all sequences in the non-redundant databases using the BLASTX program.

### Reverse Transcription Polymerase Chain Reaction Analysis

Primer sets used in the semi-quantitative RT-PCR and the number of cycles used in the amplification are indicated in Table 1. PCR amplifications were performed in 20 µL reaction mixes of 200 µM for each dNTP and 2 mM MgCl_2_, containing 0.5 units of rTaq enzyme (Takara), 1 µL of the cDNA solution obtained from each sample of plant material. The thermocycling program started with an initial 90 s denaturation step at 94°C, followed by 25, 30, or 35 cycles (30 s at 94°C, 15 s at 55°C, 90 s at 70°C) to elucidate the exponential amplification cycle for each primer combination, and final 7-min incubation at 70°C. As a control for mRNA quantity, the constitutive gene 18S rRNA was used.

**Table 1:** Primers for rt-PCR analysis.

Gene	Forward primer	Backward primer
18S rRNA	5′-GTAGTCCATGCCGTAAACGATGAGT-3′	5′-TGTCTGAGCAACACAAGACGAGGGT-3′
Tubulin A/Ftsz	5′-TCACCGACATACCAGTGAACGAATG-3′	5′-GTATGTTGTACCGTGGTGATGTCGT-3′
Phytochrome b	5′-TGAGTAAATGATGACCAGAGGCAGT-3′	5′-TTTCTCAAGCACCAAAGAGCCGTCC-3′
Aldehyde oxidase	5′-CACAATGAACCGATGACGCAACAAC-3′	5′-CTGGATACCATAAGAACCGTGTGCT-3′
TTR Protein	5′-ATCCTTGAGGATCTTGTTGTAGCTG-3′	5′-GTGCCAATCAAACTTCAACAACCAA-3′

### Data Analysis for Pathogenic Effects of *Curvularia eragrostidis* on Crabgrass

Data of weed-control effect was analysed by ANOVA using the program SPSS, procedure GLM (General Linear Model) followed by Tukey’s Test at *p*<0.05.

## Results and Discussion

### 1. Effects of *C. eragrostidis* Conidia Inoculation Concentration and Dew Duration of on Pathogenicity to Crabgrass

To assess the pathogenicity of *Curvularia eragrostidis* on crabgrass, a combination of two inoculation concentrations of *C. eragrostidis* conidia and two dew durations was performed on large crabgrass. Disease severity and differences among the treatments were evaluated by mortality and dry weight determination (Table 2).

**Table 2:** Effects of inoculation concentrations and dew durations of *Curvularia eragrostidis* on crabgrass growth.

No	Inoculation concentration(conidia/mL)	Dew duration(h)	Mortality(%)	Reduction in dry weight(%)
1	5×10^5^	12	38.5b^a^	57.66b
2	5×10^5^	24	71.59d	86.62d
3	1×10^6^	12	65.78c	79.3c
4	1×10^6^	24	89.52e	97.82e

a Values denoted by different letters are significantly different according to Tukey’s Test at *p*<0.05.

**Figure 1:**
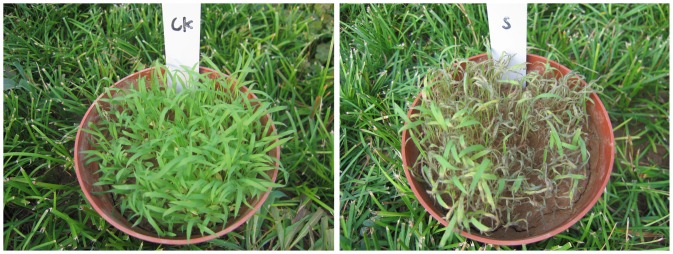
Effect of application of *Curvularia eragrostidis on Digitaria sanguinalis* grown in pots. CK: Control, sprayed with water, S: Sprayed with water containing 1×10^6^ conidia mL^−1^. The photograph was taken seven days after plants were sprayed.

Higher conidia concentration produced severe disease effects on crabgrass, At equal concentration inoculation, a longer dew duration increased disease severity to crabgrass, thus at the lower inoculation prolonging the dew duration overcame the dose effect and significantly improved the mycoherbicidal effects. Best crabgrass control was achieved with the higher inoculation concentration (with 1×10^6^ conidia/mL of *C. eragrostidis* coupled with 24 dew (Fig. 1).

### 2. Gene Expressions Under Different *Curvularia eragrostidis* Treatments

**Figure 2:**
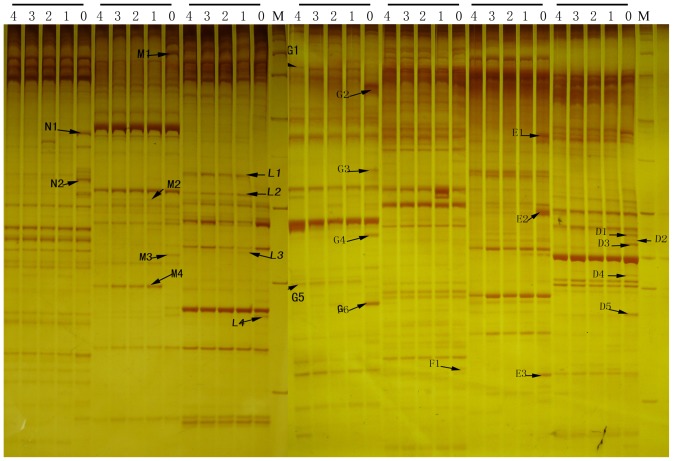
Gel electrophoresis analysis of cDNA-AFLP. M: 100 bp ladder, 0: CK, 1∼4: Treatment 1∼4 as described in Table 2, every 5 lanes are derived from selective PCR of one pair of primers.

Genome-wide cDNA-amplified fragment length polymorphism analysis allowed us to compare transcriptional changes in four combinations of inoculation concentrations and dew durations. Transcript-derived fragments displayed by cDNA-AFLP ranged in size from 100 to 1000 bp. Differential gene expression was detected for 1214 of the approximately 6,500 transcript fragments examined. Proportion of transcripts down regulated (530, 44%) and up-regulated (518, 43%) by *C. eragrostidis* treatments were very similar (Fig. 2). Therefore this fungus is able to indicates substantial change in gene expression in its host.

**Figure 3:**
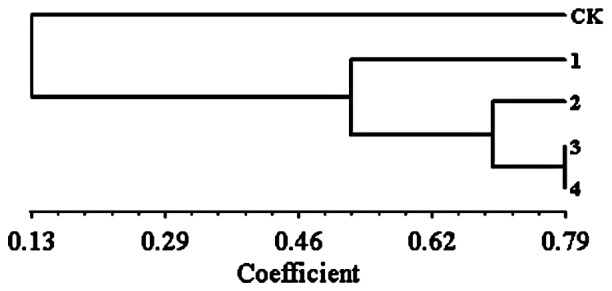
Dendrogram of effects of *Curvularia eragrostidis* on crabgrass based on UPGMA analysis of gene expression using cDNA-AFLP by NYSYS2.0. CK, 1, 2, 3, 4 same to those in Table 2

The difference of this change between the untreated check (CK) and four inoculation treatments was further evaluated using a UPGMA (Fig. 3). The similarity coefficient ranged from 0.13 to 0.79 with the CK clustering separately from the inoculation treatments. The similarity coefficient was 0.79 between treatment 3 and 4, 0.70 among treatment 2 and treatment 3 and 4, and 0.53 among treatment 1 and treatment 2, 3 and 4. The plants that diverged more from the untreated control were those inoculated with the highest dose but without any difference associated with dew duration (common similarity coefficient of 0.79). Plants inoculated with the lower conidia concentration but with the longer dew duration (treatment 2) had a slightly lower rate of induced gene expression, with a similarity coefficient of 0.70. The lowest similarity coefficient (0.53) was observed when treatment 1 (low conidia concentration, low dew duration) was compared to the rest of inoculating treatments. Therefore inoculation concentration produced more significant effects than dew duration on gene expression, and a high inoculation concentration might be crucial for better crabgrass control by using *Curvularia eragrostidis*.

### 3. Cloning and Sequencing Analysis of Differentially Expressed TDFs

**Figure 4:**
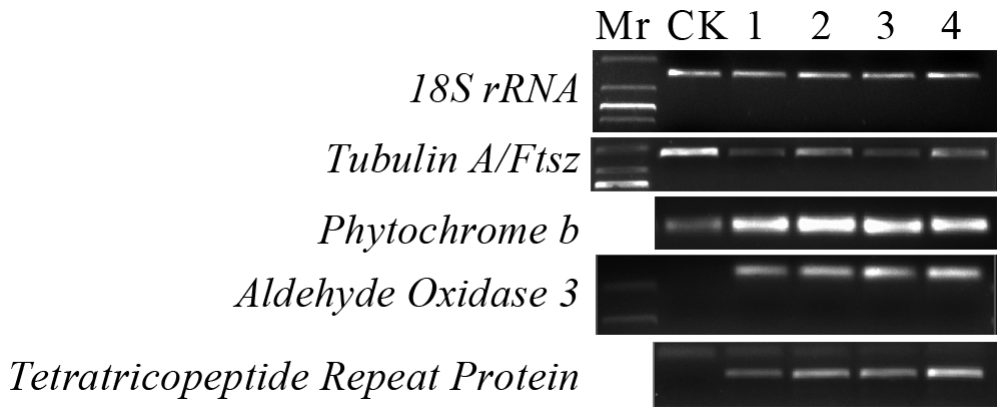
RT-PCR verification of selected TDFs.

To enable further analysis of the DE TDFs, we cloned the majority of the 46 TDFs that were consistently up- or down-regulated in *C. eragrostidis*-treated crabgrass compared to the control, including two TDFs with opposite pattern. DE TDFs were excised from gels, re-amplified, cloned, and sequenced. A total of 35 DE TDFs were successfully cloned and sequenced, 25 of them were annotated by a similarity search using the Blastx GenBank non-redundant public sequence database (Table 3). The remaining 11 DE TDFs corresponded to invalid sequences with no biological significance. To validate cDNA-AFLP data, four genes including *tubulin A/Ftsz* (24), *phytochrome b* (35), *aldehyde oxidase 3* (31), and *tetratricopeptide repeat protein* (30) were verified by reverse transcription PCR (Fig. 4). Their expression patterns corresponded to those of cDNA-AFLP results (Table 3). Therefore, Databank search revealed the relationship of these clones with genes involved in early pathogen response.

**Table 3:** Molecular cloning and sequencing analysis differentially expressed TDFs.

TDF	Primer	Clone size (bp)	Expression		Annotation	Accession number
			CK	Treat		
1	E3/M4	298	**+**	**−**	No Match	No Match
2	E3/M7	199	**+**	**−**	Proteasome Maturation Factor UMP1	XP_318683.2
3	E3/M9	112	**+**	**−**	GTP–Binding Protein	XP_311198.2
4	E4/M5	245	**+**	**−**	Vasodilator–Stimulated Phosphoprotein	XP_001654872.1
5	E4/M6	140	**+**	**−**	No Match	No Match
6	E3/M5	167	**+**	**−**	Acyl–CoA Dehydrogenase	XP_001649744.1
7	E8/M7	286	**+**	**−**	Hypothetical Protein PHYSODRAFT_509769	XP_002897274.1
8	E4/M2	181	**+**	**−**	Hypothetical Protein Aael_AAEL014423	XP_001648755.1
9	E4/M2	142	**+**	**−**	40S Ribosomal Protein S15A	NP_524709.1
10	E4/M5	143	**+**	**−**	ATP Synthase Subunit Gamma, Mitochondrial–Like	XP_003573877.1
11	E14/M6	151	**+**	**−**	No Match	No Match
12	E2/M7	128	**+**	**−**	No Match	No Match
13	E3/M6	176	**+**	**−**	Adducin	XP_001661417.1
14	E3/M4	260	**+**	**−**	No Match	No Match
15	E7/M1	142	**+**	**−**	Replication Protein A 70 Kda DNA–Binding Subunit	XP_001844125.1
16	E7/M1	132	**+**	**−**	Splicing Factor U2AF 38 kDa Subunit	XP_004926538.1
17	E7/M1	119	**+**	**−**	No Match	No Match
18	E14/M10	184	**+**	**−**	Hypothetical Protein	XP_003296749.1
19	E3/M11	74	**+**	**−**	No Match	No Match
20	E2/M12	240	**+**	**−**	Splicing Arginine Serine–Rich 6	XP_004960314.1
21	E16/M6	341	**+**	**−**	eif1_SUI1	XP_004961617.1
22	E2/M7	347	**+**	**−**	No Match	No Match
23	E3/M6	159	**↑**	**↓**	No Match	No Match
24	E4/M2	370	**↑**	**↓**	Tubulin A/Ftsz	XP_004326123.1
25	E4/M3	169	**↑**	**↓**	Leucyl–tRNA Synthetase	XP_001657794.1
26	E4/M5	115	**↑**	**↓**	Heat Shock 70 kDa Protein 4	XP_001870146.1
27	E3/M6	187	**↑**	**↓**	Photosystem II Reaction Center Protein Z–Like	XP_003571810.1
28	E5/M16	268	**↑**	**↓**	No Match	No Match
29	E3/M6	130	**↑**	**↓**	Protein Disulfide Isomerase	NP_491995.1
30	E4/M3	226	**−**	**+**	Tetratricopeptide Repeat Protein	XP_004961051.1
31	E5/M7	202	**−**	**+**	Aldehyde Oxidase 3	XP_004955959.1
32	E4/M5	185	**−**	**+**	Transmembrane 9 Superfamily Protein Member 4	XP_004965719.1
33	E5/M12	147	**−**	**+**	PutativeSenescence–Associated Protein	XP_002118266.1
34	E4/M5	143	**↓**	**↑**	ATP Synthase Gamma Chain, Mitochondrial Precursor	XP_003573877.1
35	E4/M2	152	**↓**	**↑**	Phytochrome B	XP_004984602.1

### 4. Genes and Molecular Mechanisms Involved in Early Response of Crabgrass to Inoculation of *C. eragrostidis*

Of the 35 genes identified, only six genes (17%) were up-regulated in *C. eragrostidis*-treated crabgrass. Among the up-regulated genes, aldehyde oxidase 3 and phytochrome B are two genes related to abscisic acid (ABA) biosynthesis and response. Aldehyde oxidase 3 catalyzes the last step of ABA biosynthesis in *Arabidopsis*, specifically in rosette leaves. Other aldehyde oxidases may be involved in ABA biosynthesis in other organs [18]. Activity of this enzyme can produce H_2_O_2_ [19]. Phytochrome B can enhance ABA sensitivity in *Arabidopsis* [20]. Transmembrane 9 superfamily protein member 4 (32) may be involved in stress signal transduction. A senescence–associated protein (SAP) was also up-regulated after fungal inoculation. SAPs are involved in macromolecule degradation, nutrient recycling and transport, sugar metabolism, detoxification of oxidative metabolites, and establishment of stress tolerance, several SAPs are also influenced by abiotic or biotic stresses [21]. The up-regulated tetratricopeptide repeat protein (30) is similar to *Arabidopsis* TTL1, which is related to cell redox homeostasis, and positively regulates an abscisic acid-mediated signaling pathway [22]. Taken together, Increased ABA biosynthesis coupled by enhanced ABA sensitivity may be one of the most important molecular mechanisms induced by the pathogen that mediates plant stress responses.

Most differentially expressed genes (83%) were down-regulated in *C. eragrostidis*-inoculated crabgrass, suggesting that fungus mainly negatively regulated host gene expression. The putative functions of these gene products those involved in include protein synthesis (40S ribosomal protein S15A, eif1_SUI1, leucyl–tRNA synthetase), protein modification and degradation (proteasome maturation factor UMP1, protein disulfide isomerase, heat shock 70 kDa protein 4), photosynthesis (photosystem II reaction center protein Z–Like), pre-mRNA splicing (splicing factor U2AF 38 kDa subunit, splicing arginine serine–rich 6), mitochondrial metabolism (acyl–CoA dehydrogenase, ATP synthase subunit gamma, mitochondrial–Like), DNA metabolism (replication protein A 70 Kda DNA–binding subunit), and citoskeleton (tubulin A/Ftsz, vasodilator–stimulated phosphoprotein, adducin). Plant mitochondria appeared to be somehow disfunctional, as expression of two genes (acyl–CoA dehydrogenase, mitochondrial–Like ATP synthase subunit gamma) involved in material and energy function were blocked by fungal infection. Moreover, proteasome maturation factor UMP1 was also inhibited by the pathogen; this protein was reported recently to influence the stability of the yeast mitochondrial genome, thereby leading to mitochondrial dysfunction [23], Photosystem II reaction center protein Z–Like controls the interaction of photosystem II cores with the light-harvesting antenna; attenuated expression of this protein will inevitably result in lowered photosynthesis efficiency, and thus decreased plant growth. It is interesting that a tubulin A/Ftsz was also down-regulated in pathogen-infected plants. This protein is a self-assembling GTPase similar to eukaryotic tubulin that is key for chloroplasts binary fission. Perhaps chloroplast division was also disordered [24]. Additionally, several genes including are related to reactive oxygen activity. Production of reactive oxygen species (ROS) was elevated in a UMP1 deletion mutant of *Saccharomyces cerevisiae* [23]. Protein disulfide isomerase (PDI) is a converging hub for ER-dependent ROS generation and consumption pathways. PDIs are likely to mediate at least part of its several reported functions in pathophysiology in humans, for example, apoptosis [25]. About the structure and function of PDIs in plants is very limited, one study demonstrated that PDI5 was expressed in endothelial cells about how to undergo programmed cell death (PCD) in developing *Arabidopsis* seeds [26]. Therefore, ROS homeostasis was likely broken after fungal inoculation and during fungal infection.

## Conclusions

In the present study, we compared the pathogenic and genetic effects of methods of *C. eragrostidis* stain QZ-2000 on *D. sanguinalis*. The gene expression profiles were simultaneously analyzed by cDNA-AFLP technology. High spore concentration inocubation strengths prolonging the dew duration overcame this limitation and impoved mycoherbicidal effects. *C. eragrostidis* may induce ABA biosynthesis and enhance the sensitivity to ABA. A large number of genes were inhibited or suppressed to express after inoculation. Some genes essential for mitochondria and chloroplast function were inhibited or suppressed to be expressed. Several genes related to ROS homeostasis were impacted. Thus, we conclude that infection of *D. sanguinalis* by *C. eragrostidis* distort gene expression, ABA and ROS impacting regulatory mechanisms involved in plant stress responses and plant mitochondrial and chloroplast functions. All those effects may ultimately lead to decreased plant growth or to death.

## Supporting Information

Table S1 **Selective amplification primer sequences used in cDNA-AFLP analysis.**
(DOC)
