# Supplementary material for: Differential Gene Expression for Curvularia eragrostidis Pathogenic Incidence in Crabgrass (Digitaria sanguinalis) Revealed by cDNA-AFLP Analysis
Source: PLoS One. 2013 Oct 8;8(10):e75430. doi: 10.1371/journal.pone.0075430 (PMC3792964; doi:10.1371/journal.pone.0075430)
Supplement: Table S1 — Selective amplification primer sequences used in cDNA-AFLP analysis. (DOC) [file pone.0075430.s001.doc]

**Table S1** Selective amplification primer sequences used in cDNA-AFLP analysis

| Primers for cDNA-AFLP amplification | Sequences |
| --- | --- |
| E1 | 5’-GACTGCGTACCAATTCAT-3’ |
| E2 | 5’-GACTGCGTACCAATTCAG-3’ |
| E3 | 5’-GACTGCGTACCAATTCAC-3’ |
| E4 | 5’-GACTGCGTACCAATTCTG-3’ |
| E5 | 5’-GACTGCGTACCAATTCTC-3’ |
| E6 | 5’-GACTGCGTACCAATTCGC-3’ |
| E7 | 5’-GACTGCGTACCAATTCTA-3’ |
| E8 | 5’-GACTGCGTACCAATTCGA-3’ |
| E9 | 5’-GACTGCGTACCAATTCCA-3’ |
| E10 | 5’-GACTGCGTACCAATTCGT-3’ |
| E11 | 5’-GACTGCGTACCAATTCCT-3’ |
| E12 | 5’-GACTGCGTACCAATTCCG-3’ |
| E13 | 5’-GACTGCGTACCAATTCAA-3’ |
| E14 | 5’-GACTGCGTACCAATTCTT-3’ |
| E15 | 5’-GACTGCGTACCAATTCCC-3’ |
| E16 | 5’-GACTGCGTACCAATTCGG-3’ |
| M1 | 5’-GATGAGTCCTGAGTAAAT-3’ |
| M2 | 5’-GATGAGTCCTGAGTAAAG-3’ |
| M | 5’-GATGAGTCCTGAGTAA AC-3’ |
| M4 | 5’-GATGAGTCCTGAGTAA TG-3’ |
| M5 | 5’-GATGAGTCCTGAGTAA TC-3’ |
| M6 | 5’-GATGAGTCCTGAGTAA GC-3’ |
| M7 | 5’-GATGAGTCCTGAGTAA TA-3’ |
| M8 | 5’-GATGAGTCCTGAGTAAGA-3’ |
| M9 | 5’-GATGAGTCCTGAGTAACA-3’ |
| M10 | 5’-GATGAGTCCTGAGTAAGT-3’ |
| M11 | 5’-GATGAGTCCTGAGTAACT-3’ |
| M12 | 5’-GATGAGTCCTGAGTAACG-3’ |
| M13 | 5’-GATGAGTCCTGAGTAAAA-3’ |
| M14 | 5’-GATGAGTCCTGAGTAATT-3’ |
| M15 | 5’-GATGAGTCCTGAGTAACC-3’ |
| M16 | 5’-GATGAGTCCTGAGTAAGG-3’ |

Primers pairs for cDNA-AFLP amplification were E1-16×M1-16,the total was 256 primers pairs.
